# (No) Effects of a Self-Kindness Intervention on Self-Esteem and Visual Self-Perception: An Eye-Tracking Investigation on the Time-Course of Self-Face Viewing

**DOI:** 10.3390/ejihpe13110179

**Published:** 2023-11-08

**Authors:** Jonas Potthoff, Anne Schienle

**Affiliations:** Department of Psychology, University of Graz, 8010 Graz, Austria; anne.schienle@uni-graz.at

**Keywords:** self-face, self-kindness, self-esteem, depression, smartphone intervention, app-assisted approach, eye-tracking

## Abstract

Previous research has suggested a favorable impact of self-kindness on subjective well-being. The present experiment investigated the effects of an app-assisted self-kindness intervention for increasing self-esteem and self-face gaze, and for decreasing depression. We explored self-face processing via a time-course analysis of eye-tracking data. Eighty participants (56 female, 24 male; mean age: 23.2 years) were randomly allocated to one of two intervention groups, each receiving daily instructions to enhance either self-kindness or relaxation (active control). Following a one-week intervention period, both groups reported improved self-esteem (*p* = .035, *η_part_*^2^ = .068) and reduced depression (*p* < .001, *η_part_*^2^ = .17). The duration of self-face gaze increased in both groups (*p* < .001, *η_part_*^2^ = .21). Self-face processing was characterized by an early automatic attention bias toward the self-face, with a subsequent reduction in self-face bias, followed in turn by an attentional self-face reapproach, and then a stable self-face bias. We thus identified a complex temporal pattern of self-face inspection, which was not specifically altered by the intervention. This research sheds light on the potential for app-assisted interventions to positively impact psychological well-being, while also highlighting the complexity of self-face processing dynamics in this context. In the future, we propose the inclusion of personalized self-kindness statements, which may amplify the benefits of these interventions.

## 1. Introduction

How do people perceive and treat themselves during moments of self-reflection? Is their behavior characterized by self-criticism and harsh judgment, or by patience, compassion, and self-kindness? Self-kindness is described as the inclination to demonstrate care and understanding towards oneself, especially during periods of heightened stress [1]. Self-kindness serves as a resource for maintaining a positive self-image when grappling with perceived shortcomings or personal challenges [2].

Self-kindness can be effectively enhanced through self-affirmation techniques [3]. Self-affirmation theory, postulated by Steele [4], posits that people are inherently driven to preserve a positive self-image. For example, if encountering a threat to one’s self-image, one’s response may often include an effort to restore the positive self-image, such as by validating one’s positive attributes in a friendly and affirming manner; this effectively compensates for perceived shortcomings [5]. In a study by Lannin et al. [6], adolescents who engaged in positive self-affirmation of their strengths during challenging situations reported elevated self-esteem four months later. However, it is important to distinguish between positive self-affirmation in general and the more specific concept of self-kindness. With the latter, the focus is not only on acknowledging one’s positive attributes but also on recognizing weaknesses with compassionate honesty, with a lack of excessive self-criticism [7].

Self-affirmation and self-kindness are nonetheless closely related: one approach for enhancing self-kindness is indeed the practice of repeating affirmative self-statements. In a study by Philpot and Bamburg [8], participants repeated positive statements, such as “I look good and I feel happy. Things are great” three times every day. After two weeks, participants reported heightened self-esteem and reduced depression compared to the control group. It is important to note that excessively optimistic self-statements, such as “I am the most beautiful person in the world”, may, in some cases, prove counterproductive and foster self-doubt and depressed mood instead of bolstering self-esteem [9].

So far, evidence on the efficacy of self-directed kindness statements in promoting self-esteem and alleviating depression is sparse and contradictory [8,10]. Importantly, these studies have relied on retrospective assessments, wherein participants were required to recall the daily frequency of using self-statements [10]. This recall approach may, however, introduce biases, as participants might remember only those self-statements that had yielded positive results, or only those that had been most recently employed. Consequently, the current study used a smartphone-guided self-kindness approach to investigate the effects of a self-kindness intervention on self-esteem and depression. This app-assisted approach eliminated potential recall biases. Moreover, mobile app self-kindness interventions present a promising method for reaching a large number of people, as well as being easy to implement into one’s daily routine [11,12].

In the present study, a smartphone app verbally guided participants, via prerecorded auditory instructions, to recite self-directed kindness statements three times a day for one week. Further, for the first time, we investigated the effects of self-kindness practice on self-face viewing via eye-tracking. In the context of interventions that focus on self-kindness and self-esteem, eye-tracking data can provide valuable insights into less conscious self-perception processes that may not be captured by explicit information provided by participants (e.g., via questionnaires). Previous eye-tracking studies have shown that a more negative self-image is associated with self-face avoidance when image pairs of self-face and unknown faces are freely viewed. In contrast, a more positive self-image (i.e., high self-esteem) has been shown to increase visual attention to one’s own face [13,14,15]. The studies cited here have used brief exposure times (≤5 s). However, if participants do not have sufficient time to view both faces, the possibility of differentiating between different attentional processes (e.g., automatic vs. controlled/prolonged attention) might be restricted. Some studies with free-viewing paradigms have shown that gaze behavior becomes more and more controlled over time (i.e., with longer exposure durations; [16]). For example, Ypsilanti, Robson, et al. [15] found that a more negative self-image (i.e., high self-disgust) did not affect early automatic self-face processing (within the first second) but was, however, associated with conscious gaze avoidance of one’s face (within the subsequent four seconds). A recent eye-tracking study [17] implemented a (much) longer duration of self-face exposure. Participants in that study were exposed to their faces in a mirror for 90 s. A more negative self-image (i.e., lower self-esteem) was associated with longer, possibly more critical, self-face viewing. These findings collectively suggest time-dependent factors involved in self-face viewing, which necessitate the nuanced differentiation between early automatic and later, more consciously controlled gaze behaviors. Thus, the current experiment included a time-course analysis of eye-tracking data to investigate the gaze behavior of participants in the context of the self-kindness intervention.

In the present study, participants were presented with pairs of images depicting their own faces (self-faces) alongside the faces of unknown others (other-faces). Participants were randomly allocated to either a self-kindness intervention group or a relaxation control group, both lasting a period of one week. Assessments of self-face viewing behavior, self-esteem (Multidimensional Self-Esteem Scale, MSES [18]), and depression (Brief Symptom Inventory 18, BSI-18; [19]) were conducted before and after the intervention.

The following hypotheses were preregistered in the Open Science Framework (OSF: https://osf.io/sj4bx/; reregistered on 26 of February 2021):

1. The self-kindness intervention (compared to the control intervention) will increase self-esteem [6,8].
2. The self-kindness intervention (compared to the control intervention) will increase gaze duration for the self-face.

Additionally, we explored the effects of the intervention on depression, as well as how self-face viewing changes over time, from early, automatic self-face viewing to later, more consciously controlled viewing [15].

## 2. Materials and Methods

### 2.1. Participants

A statistical power analysis for the within–between interaction was performed for sample size estimation. Based on a medium-size effect of *η_part_*^2^ = .06 (*α* = .05, power = .95), the projected sample size needed with this effect size (G*Power 3.1.9.7; [20]) was *n* = 54. Assuming some missing data due to insufficient calibration (i.e., eye-tracking data) and insufficient compliance (i.e., smartphone app compliance), we recruited a total of 80 adult participants (56 female, 24 male) with a mean age of 23.2 years (*SD* = 3.64 years, range: 18 to 36 years) to participate in this study. None of the participants reported somatic illnesses or mental disorders (self-reported in an online prescreening). Most participants were university students (71 university students, 8 white-collar workers, and 1 unemployed). Participants had normal or corrected-to-normal vision. Only participants who used the smartphone app for at least four of the seven days were included in the data analysis (*n* = 65). The participants were randomly assigned to the self-kindness intervention (*n* = 34) or the relaxation intervention (*n* = 31). The groups did not differ in age and gender (*p_Holm_* > .06).

### 2.2. Procedure

Both groups used the app to receive auditory instructions comparable in duration and frequency. We chose an active control group (instead of a waiting-list group) to avoid overestimation of treatment effects [21]. Before and after the one-week intervention, questionnaires and eye-tracking data were assessed. At the end of the session before the intervention, the smartphone app was installed. The day after the lab session, participants started the one-week app-assisted intervention. In the second lab session (seven days later), questionnaires and gaze data were assessed again.

### 2.3. Questionnaires

Self-esteem was assessed via the Multidimensional Self-Esteem Scale (MSES; [18]), which consists of 32 items (e.g., “How often are you satisfied with yourself?”) rated on a 7-point Likert-scale from one (intensity: “not at all”/frequency: “never”) to seven (intensity: “very much”/frequency: “always”), with 23 out of the 32 items being reverse-scored (i.e., “very much”/”always” would indicate the lowest self-esteem and would be scored as a one). The MSES distinguishes between five subscales (domain specific self-esteem in the domains: social contact, social criticism, performance, physical appearance, and physical ability) and global self-esteem. In the present sample, for global self-esteem, the Cronbach’s alpha was .95 before and .96 after the intervention. Due to the high internal consistency, we refer to global self-esteem with mean values of one indicating the lowest possible self-esteem and seven indicating the highest self-esteem assessable by the MSES.

Symptoms of depression were assessed with the depression scale of the German 18-item version of the Brief Symptom Inventory (BSI-18; [19]). The BSI-18 distinguishes between the three subscales: depression (e.g., “How much have you been suffering from sadness during the last seven days?”), somatization, and anxiety (six items per subscale; no inverted items; all item responses scored on a 5-point Likert scale from zero = “not at all” to four = “very strongly”). The six items of each BSI-18 subscale are aggregated to a sum score (i.e., from 0 to 24). In the present sample, for the depression scale, the Cronbach’s alpha was .74 before and .76 after the intervention.

### 2.4. Eye-Tracking

During eye-movement recordings, participants freely viewed (compare [22]) image pairs of self-face and other-face. Six other-faces with neutral facial expressions were retrieved from the FACES database [23,24]. The stimuli were faces of actors similar in age and the same gender as the participants (other-faces female actresses: 034, 048, 115, 125, 140, 152; other-faces male actors: 081, 114, 127, 144, 167, 175). At the beginning of each laboratory session, a self-face picture with a neutral facial expression was taken in front of a grey background, with the participant wearing a neutral (i.e., grey) t-shirt to match the visual characteristics of the other-faces stimuli as closely as possible. The stimuli were presented as image pairs side by side on a white background. Each image (six other-faces, one self-face) was displayed in an image pair with each other image twice (resulting in 84 trials). The second time an image pair was presented, the arrangement (which image was on the left or right side of the screen) was mirrored. All image pairs of two other-face images were shown to present every face in the same number of trials. However, only the gaze data of trials with the self-face present were analyzed. Images were presented in a size of 600 by 800 pixels. Before each trial (trial duration = 10 s), participants had to fixate on a fixation cross in the center of the screen for 500 ms. The trial order was randomized.

During the eye-movement recording, a chin rest minimized head movements and stabilized the head at a viewing distance of 60 cm. An eye-tracker (SMI RED 250 mobile) recorded two-dimensional eye movements at a sampling rate of 250 Hz. Both eyes were calibrated and recorded, but we analyzed data from the eye with a better calibration (typically a < 0.35° visual angle). Images were presented on a white background on a 24″ screen (1920 × 1080 pixels). The experiment was controlled via SMI Experiment Center (Version 3.6.53). The velocity threshold for saccade detection was 40°/s. The absence of saccades and blinks defined fixations (minimum fixation duration = 50 ms). Data were exported using SMI BeGaze. Self-face and other-face images were defined as rectangular areas of interest (AOI). We conducted gaze data analysis exclusively for AOIs of trials with self-face and other-face image pairs.

We computed the percentage of gaze duration (i.e., total fixation duration) spent on the self-face (e.g., 58% would indicate that 58% of the gaze was spent on the self-face and 42% on the other-face). For the time-course analysis, we computed the percentage of fixations for ten 1 s intervals throughout the trial to investigate changes in the self-face processing over time. Fixations were assigned to the ten intervals based on their time of onset.

### 2.5. Smartphone App

A self-designed smartphone app (running on Android and iOS) provided the auditory instructions to practice self-statements. Participants installed the app on their smartphones at the end of the first laboratory session. On each day of the intervention, upon opening the app, participants rated their valence and arousal on scales from one to nine (one = negative, calm; nine = positive, aroused). Subsequently, a voice presented the statement of the day. Participants could choose between a male and a female voice for the auditory instructions. The app introduced the self-statement and instructed the participants to repeat each statement three times (e.g., self-kindness: “I am enough”, “I am precious”; relaxation: “I am calm”, “My breathing is calm and regular”). After introducing the sentence and rehearsing the statement three times, participants rated valence and arousal again. Participants were asked to use the app at least once daily in an undisturbed, quiet environment. Only ratings before and after the completed self-statement instructions (i.e., when the auditory instruction had been played entirely) were analyzed. If participants conducted the self-statement less than four out of the seven days, their data were excluded from the analyses. Both conditions contained seven different statements (one per day), and their order was randomized for each participant individually. Hence, all participants (who completed all seven statements) of the same condition practiced the same seven statements.

## 3. Results

### 3.1. Statistical Analysis

We computed 2 × 2 analyses of variance (ANOVA) to test the effects of intervention (between subjects: self-directed kindness and relaxation) and time (within-subjects: before the intervention and after the intervention) on valence, arousal, self-face viewing (i.e., gaze duration percentage), self-esteem, and depression. For the time-course analysis, we calculated an exploratory 2 × 2 × 10 ANOVA, with the additional within-subject factor interval (1st to 10th second of the trial). If assumptions of sphericity were violated, Greenhouse–Geisser corrected results are reported.

### 3.2. Valence and Arousal

For valence, there was a significant effect of time (before vs. after treatment); *F*(1,63) = 13.12, *p* < .001, *η_part_*^2^ = .17; which resulted from more positive ratings after the intervention (pre: *M* = 5.87, *SD* = 1.33; post: *M* = 6.22, *SD* = 1.35). Neither the main effect of intervention; *F*(1,63) = 0.02, *p* = .90, *η_part_*^2^ < .001; nor the interaction were significant; *F*(1,63) < 0.01, *p* = .97, *η_part_*^2^ < .001. Also, for arousal, only the main effect of time was significant; *F*(1,63) = 61.04, *p* < .001, *η_part_*^2^ = .49; while the effect of intervention; *F*(1,63) = 2.24, *p* = .14, *η_part_*^2^ = .034; and the interaction were not; *F*(1,63) = 0.58, *p* < .45, *η_part_*^2^ = .009. The effect of time was caused by a decrease in arousal after the intervention (pre: *M* = 2.67, *SD* = 1.19; post: *M* = 2.07, *SD* = 0.86).

### 3.3. Gaze Duration Percentage

The main effect of time on self-face gaze duration percentage was significant: *F*(1,59) = 15.18, *p* < .001, *η_part_*^2^ = .21. Neither the main effect of intervention; *F*(1,59) = 0.93, *p* = .34, *η_part_*^2^ = .015; nor the interaction was significant; *F*(1,59) < 0.001, *p* = .97, *η_part_*^2^ < .001. The main effect of time resulted from a higher gaze duration that was spent on self-faces after the intervention: *t*(60) = 3.93, *p* < .001, *d* = 0.50 (Table 1).

### 3.4. Self-Esteem

There was a significant main effect of time on self-esteem: *F*(1,63) = 4.63, *p* = .035, *η_part_*^2^ = .068. Neither the main effect of intervention; *F*(1,63) = 0.84, *p* = .36, *η_part_*^2^ = .013; nor the interaction were significant; *F*(1,63) < 0.001, *p* = .98, *η_part_*^2^ < .001. The main effect of time resulted from higher self-esteem after the intervention: *t*(64) = 2.17, *p* = .034, *d* = 0.27 (Table 1).

### 3.5. Depression

The main effect of time was significant for depression; *F*(1,63) = 12.58, *p* < .001, *η_part_*^2^ = .17. Overall, depression was lower after the intervention (Table 1); *t*(64) = 3.60, *p* < .001, *d* = 0.45. The effect of intervention; *F*(1,63) = 1.69, *p* = .20, *η_part_*^2^ = .03; and the interaction were not significant; *F*(1,63) = 0.63, *p* = .43, *η_part_*^2^ = .01.

### 3.6. Time Course of Self-Face Fixation Percentage

**Figure 1:**
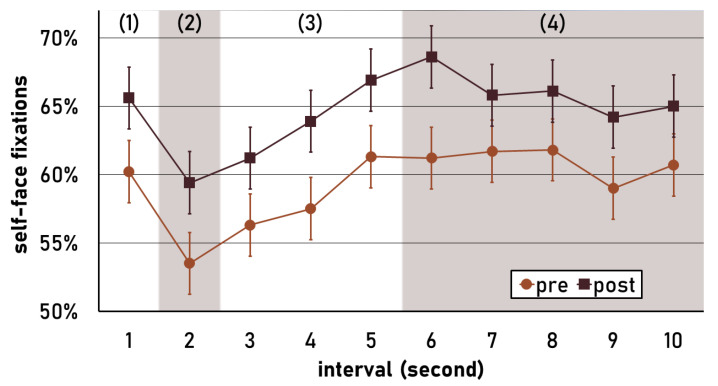
Estimated marginal means of self-face fixation percentage over the course of ten one-second intervals. A percentage above 50 indicates that more time was spent at the self-face compared to the other-face. Pre: before intervention. Post: after intervention. Error bars indicate pooled standard errors. (1): Automatic self-face bias, (2): reduction in self-face bias, (3) redirected attention to self-face, (4) stable self-face bias.

For the exploratory self-face fixation percentage, there was a significant effect of interval; *F*(9,513) = 3.50, *p* < .001, *η_part_*^2^ = .058; and time (pre/post-intervention); *F*(1,57) = 18.23, *p* < .001, *η_part_*^2^ = .242. The effect of interval was caused by a self-face direction bias within the first second of the trial (*M* = 63.5%, *SD* = 14.3%), which decreased significantly in the second interval (*M* = 55.7%, *SD* = 16.0%): *t*(59) = 2.83, *p* = .006, *d* = 0.365. Self-faces were then reapproached until the second half of the trial (i.e., 6th interval; *M* = 64.7%, *SD* = 15.6%): *t*(59) = 4.07, *p* < .001, *d* = 0.525). During the second half of the trial self-face fixation percentage remained at an average level of 63.0% (*SD* = 11.2%). The effect of time resulted from an overall higher self-face fixation percentage after the intervention, which showed for the entire trial duration (see Figure 1). All other effects were not significant (*ps* > .11).

## 4. Discussion

The present eye-tracking study examined the effect of an app-assisted self-kindness intervention on self-esteem, depression, and self-face viewing. The self-directed kindness intervention was compared with a relaxation intervention (autogenic training: a relaxation intervention based on self-regulation; [25]). As intended, both conditions were comparable in affective ratings (i.e., increase in valence, decrease in arousal). Contrary to the hypothesis, the two interventions did not elicit differential changes in self-esteem and self-face gaze duration. Depression was also decreased by the self-directed kindness intervention and in the active control condition.

The autogenic training instructions implemented in the control group app may have positively influenced self-perception, possibly rooted in self-care via relaxation [26,27]. Enhanced relaxation and reduced stress levels could potentially contribute to less harsh self-judgment. This interpretation aligns with recent research highlighting relaxation as a potential self-esteem booster [26,27]. Consequently, future investigations on self-kindness interventions should consider incorporating multiple comparison groups, featuring varying degrees of relaxation, to shed light on the interplay between self-compassion and relaxation.

Another possible explanation for the increased self-esteem, self-face viewing, and decreased sad mood observed in both groups could be related to the initial characteristics of the participants. We investigated individuals with relatively high self-esteem and low levels of depression. Within this particular sample, engaging with oneself, whether through practices like self-kindness or autogenic training, might have enhanced their existing positive self-assessment.

In this study, we employed a self-directed kindness intervention featuring standardized statements. This approach was chosen to facilitate the assessment of an intervention that was both straightforward and broadly applicable, akin to standardized relaxation statements. While individually tailored self-statements might have potentially enhanced intervention effectiveness, they could also introduce complexity and implementation challenges for smartphone-assisted interventions for a broad audience. Encouragingly, the reported increase in valence following daily kindness interventions indicated that this approach was well-received and devoid of adverse effects, aligning with previous research [9]. However, subsequent studies should investigate whether self-chosen self-statements yield larger effects and whether interventions utilizing such statements are even less aversive than standardized counterparts.

A distinct temporal pattern characterized visual self-face processing in the present study. Initially, participants exhibited an automatic attention bias towards their own faces. Subsequently, this bias decreased in favor of other-face viewing. After this decrease, the attention was redirected towards the own face over the subsequent seconds. Ypsilanti, Robson, et al. [15] found a similar pattern from the 2nd second to the 3rd second of their trials, when presenting self- and other-face image pairs for five seconds. In the present study, during the second half of the trial (6th to 10th second), the percentage of fixations spent on the own face remained relatively stable. This sustained self-face bias in late conscious visual attention was not observable in previous research, due to the shorter exposure duration [13,14,15].

The described temporal pattern of gaze behavior was consistent across both intervention groups. Thus, we identified a general tendency to look at self-faces during the early automatic processing of faces. The following reduction in this self-face bias may represent a more controlled evaluation of the context in which the self-face is presented. Here, the own face may be compared to the other face. This evaluation of the face context is completed after a couple of seconds, and then the focus of attention is redirected towards the self-face. The temporal analysis of the eye-tracking data revealed that when looking at one’s face, one cannot merely distinguish between early (automatic) and later (controlled) processing. Rather, gaze behavior seems to be divided into (1) an early automatic self-face bias, (2) a context exploration period, and (3) a later controlled attentional redirection and evaluation of one’s face, followed by (4) temporally stable self-face attention. This observation has implications for the design of future studies, particularly in cases where faces are presented only briefly, as the full spectrum of self-face processing may not be adequately captured due to the limited exposure time (e.g., [13]).

In conclusion, the present study showed an increase in self-esteem and self-face viewing, accompanied by a decrease in depression, in response to using an app-assisted intervention for one week. These changes were present irrespective of whether the app involved self-directed kindness or relaxation. Furthermore, the current findings underscore the importance of considering the temporal dynamics of self-face processing, because multiple distinct phases characterize self-face processing, as it encompasses distinct phases of processing.

Some limitations must be acknowledged. We investigated self-face processing and self-esteem in a sample of predominantly female participants, of which most were university students. Therefore, our findings may not be generalizable to other populations or stimuli (e.g., depictions of the whole body). We investigated healthy participants with low levels of depression and moderate-to-high levels of self-esteem. Greater effects of the kindness intervention can be expected in samples with lower self-esteem. However, for people with very low self-esteem or severe (periods of) depression (e.g., patients with major depressive disorder or bipolar disorder), it needs to be evaluated if self-statements have to be adapted in order to avoid a large gap between optimistic self-statement and pessimistic self-concept [9]. Future investigations with clinical samples should take into account potential discrepancies between self-perception and self-statements and the need for a longer intervention duration than one week. This consideration will enable a more precise alignment between the content of self-statements and the timing/duration of self-kindness interventions in populations with affective disorders.

## Figures and Tables

**Table 1:** Descriptive data for gaze duration, self-esteem, and depression.

	Before	After
**Self-face gaze duration (%)**		
overall	59.9 (13.2)	65.4 (10.9)
self-kindness	58.6 (14.6)	64.1 (9.9)
control	61.2 (11.6)	66.8 (11.8)
**Self-esteem**		
overall	4.54 (0.91)	4.64 (1.04)
self-kindness	4.44 (0.92)	4.53 (1.08)
control	4.66 (0.89)	4.75 (1.01)
**Depression**		
overall	4.03 (3.24)	2.92 (2.93)
self-kindness	4.53 (3.18)	3.21 (3.29)
control	3.39 (3.16)	2.55 (2.43)

Note: Means and standard deviations (in parentheses) of duration of self-face gaze (%), self-esteem (possible range of mean score: 1–7; higher values indicate higher self-esteem), and depression (possible range of sum score: 0–24; higher values indicate higher depression) before and after intervention for the overall group and split by group: kindness = self-directed kindness group, control = active control (autogenic training) group.

## Data Availability

Data are available in the Open Science Framework (OSF) project: https://osf.io/sj4bx/ (uploaded on 14 January 2022).
